# Spontaneous reactivation of hepatitis B virus replication in an HIV coinfected patient with isolated anti-Hepatitis B core antibodies

**DOI:** 10.1186/1743-422X-11-9

**Published:** 2014-01-21

**Authors:** Rongjuan Pei, Sebastian Grund, Jens Verheyen, Stefan Esser, Xinwen Chen, Mengji Lu

**Affiliations:** 1 Wuhan Institute of Virology, Chinese Academy of Sciences, Wuhan 430071, China; 2 Institute of Virology, University Hospital of Essen, University Duisburg-Essen, Essen, Germany; 3 Institute for Virology, University of Düsseldorf, Düsseldorf, Germany; 4 Clinic of Dermatology, University Hospital Essen, University Duisburg-Essen, Essen, Germany

**Keywords:** HBV, HIV, Anti-HBc only, Reactivation, Mutation

## Abstract

Co-infections with HBV (hepatitis B virus) occur in HIV (human immunodeficiency virus) patients frequently. It has been reported that an effective treatment of HIV can also lead to a suppression of HBV and to anti-HBs seroconversion in HBV-infected patients. Here, we report a spontaneous reactivation of HBV replication in an HIV-infected patient with anti-HBc as the only marker of chronic HBV infection. The patient was known to be coinfected with HIV and HBV for years and the HBV DNA was measured repeatedly at low levels. A significant increase of HBV DNA up to 1.7x10^7^ IU/ml was found accompanied with clinical symptoms of hepatitis. Multiple mutations occurred in the S gene during the flare-up of HBV as shown by sequencing, including I103T, K122R, M133I, F134V, D144E, V164E and L175S. Anti-HIV/HBV treatment led to a resolution of symptoms and to a decrease in the HIV RNA and HBV DNA viral load. It is possible that the accumulated mutations during HBV replication were selected and responsible for the reactivation.

## Introduction

The hepatitis B virus (HBV) is a double-stranded DNA virus belonging to the family of *Hepadnaviridae,* affecting an estimated 350 million chronically infected individuals worldwide. Due to the similar transmission routes, HBV and human immunodeficiency virus (HIV) coinfection is common and an estimated 5%-15% of HIV infected patients also have HBV infection [1]. HIV coinfection increases the risk of HBV chronicity and HBV reactivation as well as the risk for the development of liver cirrhosis and hepatocellular carcinoma (HCC) [2,3].

Reactivation of a former HBV infection can occur spontaneously or triggered by immunosuppressive therapy, immunocompromising diseases, organ transplantation or withdrawal of antiviral drugs [4-8]. HBV precore mutation has been reported to be associated with spontaneous reactivation of HBeAg positive chronic hepatitis B [9]. The recurrence of HBV replication in HIV/HBV-coinfected patients has been described due to the interruption of lamivudine therapy, due to resistance to the drug [10,11] and due to HBV immune-escape or precore mutants [11-13]. However, little is known about the molecular characteristics of HBV that is reactivated spontaneously in HBV/HIV coinfected individuals. Previously, HBsAg immune escape mutants were described in the chronic phase and a flare-up phase of HBV infection of an HBV/HIV infected person [14]. Here we analyzed the HBV sequence changes in an HBV/HIV coinfected patient who suffered from a spontaneous reactivation of HBV.

## Methods

### Serology

Serum samples were stored at -70°C before analysis. Serological markers of HBV infection were determined using commercial enzyme immunoassay kits (Abbott Laboratories, IL, USA) and confirmed partly with other assays (Roche Diagnostics GmbH, Mannheim, Germany; Dade Behring GmbH, Marburg, Germany). The HBV DNA level was quantified using a commercial real-time fluorescence quantitative kit (Roche) and Versant HBV bDNA assay kit (Siemens).

### DNA extraction from sera, PCR amplification, cloning and sequencing of PCR fragments

The HBV DNA was extracted from patient sera using QIAamp DNA blood mini kit (Qiagen, Hilden, Germany) and subjected to PCR amplification using the high fidelity Taq polymerase (Roche) according to the manufacturer’s instruction. The region encoding the HBsAg (nt 2818–888) was amplified using primers FS-S1 5′-GTCACCATATTCTTGGGAAC-3′ (nt 2818–2837) and FS-AS 5′-CATATCCCATGAAGTTAAGG-3′ (nt 888–869) according to the reference sequence AY220698 and cloned into pCR2.1 vector. Five clones of each sample were sequenced.

## Results

### Clinical features

**Figure 1:**
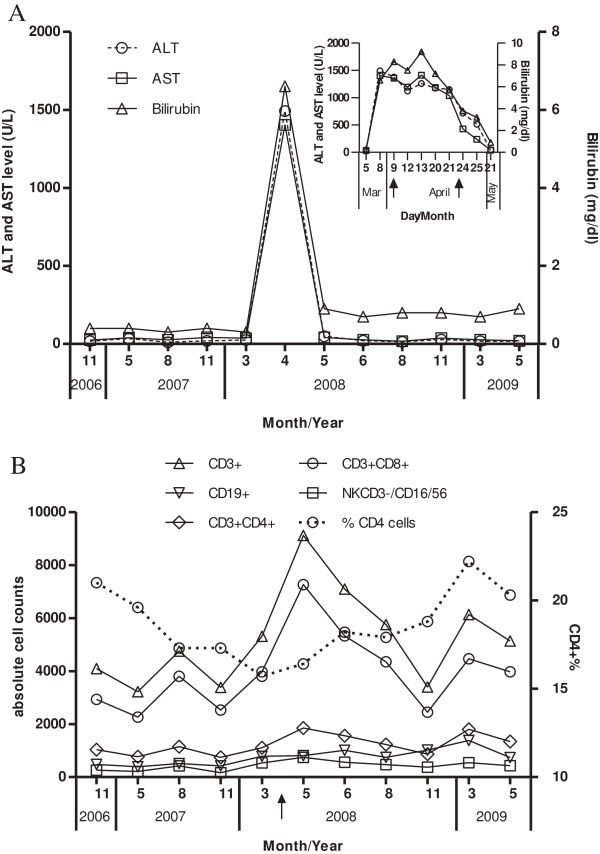
**The ALT, AST, bilirubin levels and lymphocytes counts in an HBV/HIV coinfected patient experiencing a spontaneous HBV reactivation. (A)** The ALT, AST and bilirubin levels were monitored regularly from 2006 to 2009. The ALT and AST levels were plotted at the left Y-axis and the bilirubin level at the right Y-axis. The insert indicated the ALT, AST and bilirubin levels during the flare-up phase, the black arrows indicated the sampling time for sequencing. **(B)** The lymphocytes counts were monitored regularly from 2006 to 2009. The relative CD4+ T cell counts in the CD3+ T cell population (CD4 +%) was set at the right Y-axis. The black arrow indicated the start of the antiviral therapy.

A 60-year-old patient was known to be infected with HIV-1 since 1984. He was diagnosed with a HBV coinfection in July 2003 by HBV serology (HBsAg positive, HBeAg positive and anti-HBc positive) and detectable HBV DNA in the serum. Between October 2003 and August 2007 the HBV DNA fluctuated between 10 to 357 IU/ml while the serological markers of HBV except anti-HBc were negative (Table 1). Since the patient had no HIV-associated symptoms and stable numbers of CD4 positive T helper cells > 500/μl (Figure 1B) with a relatively low HIV viremia (< 100,000 copies/ml) he did not receive a highly active antiretroviral therapy (HAART).

**Table 1:** Sequential serological and virological findings

**Month/year**	**07.2003**	**10.2003**	**08.2004**	**07.2005**	**11.2005**	**08.2006**	**08.2007**	**04.2008**	**06.2008**	**08.2008**	**11.2008**
HBsAg	+	-	-	-	-	-	+	+	ND	+	-
Anti-HBs	ND	-	-	-	-	-	-	-	ND	+	+
HBeAg	+	ND	ND	ND	-	ND	ND	+	ND	ND	-
Anti-HBe	-	ND	ND	ND	-	ND	ND	-	ND	ND	-
Anti-HBc	+	+	+	+	+	+	+	+	ND	+	+
Anti-HBc-IgM	+	-	-	ND	-	ND	ND	+	ND	-	-
HBV DNA	+	-	ND	+	+	ND	+	+		ND	-
	1.1 × 10^5^	ND	ND	357	357	ND	1009	1.7 × 10^7^	357	ND	<357
HIV	2.6 × 10^4^	1.3 × 10^4^	ND	1.5 × 10^4^	1.2 × 10^4^	1.2 × 10^4^	2.9 × 10^3^	5.3 × 10^4^	1.2 × 10^2^	ND	<50

ND means not detected.

In April 2008 the patient was admitted to hospital due to an acute icteric hepatitis with elevated serum transaminases (AST and ALT > 1000 U/mL) and cholestasis (total bilirubin more than 9.2 mg/dl) (Figure 1A). HBV serologiy (HBsAg, HBeAg and anti-HBc positive) and PCR (HBV DNA >17.86 million IU/ml) showed an exacerbation of chronic HBV infection. At the same time the HIV viral load was 53,000 copies/ml (Table 1). The presence of a co-infection with hepatitis D virus (HDV) was excluded serologically.

The patient was treated with HIV/HBV-active therapy (emtricitabine 200 mg, tenofovir 245 mg (Truvada ®) 1-0-0 , lopinavir 200 mg, ritonavir 50 mg (Kaletra ®) 2-0-2 ), and hereby a rapid virological response was achieved with HBV and HIV viremia decreasing below the limits of detection. The response to antiviral therapy was accompanied by a clinical improvement of the patient, a normalization of the transaminases and of the cholestasis parameters. Anti-HBs seroconversion was achieved 7 months later and was preceded by a phase of simultaneous detection of HBsAg and anti-HBs at 4 months after treatment as it is seen often in clinical routine. Interestingly in the long term anti-HBs declined below the detection limit and anti-HBc again remained the only positive serological HBV marker. During the entire course no significantly change of CD3 + CD4+ T-lymphocytes and the NK CD3-/CD16/56 was found; the counts of CD3 + CD8+ and total CD3+ T-lymphocytes was elevated temporarily after the HAART treatment was started, and the counts of CD19+ lymphocytes was at low level likely due to a splenectomy conducted previously due to a trauma.

### HBV mutations in the S and the Pol genes

HBV DNA fragments were amplified from 5 serum samples, three of which were collected in the early chronic phase of infection and two were collected during the flare-up phase, while other samples were negative in the HBV PCR due to the low HBV viral loads. A HBV genotype A HBsAg subtype adw2 strain was found in the chronic phase and the HBV population was homogenous at the time of the first diagnosis of HBV infection. However, the HBV population during the flare-up phase was found to be heterogeneous with multiple amino acid (aa) substitutions within the HBsAg a-determinant. Particularly, the subtype determinant aa 122 K was replaced by R gradually in the flare-up samples. More aa substitutions were found, including I103T, M133I, F134V, D144E, V164E and L175S in the HBsAg (Table 2) and T45S, N122D, V133G/N and W144G in the HBV RT sequence (Table 3). In contrast, the HBV preC/C region was also sequenced and no mutation was found during the same time period.

**Table 2:** Characterization of HBsAg sequences

		**92**	**103**	**122**	**133**	**134**	**144**	**155**	**160**	**164**	**175**	**187**
		**I**	**I**	**K**	**M**	**F**	**D**	**S**	**K**	**V**	**L**	**S**
2003.07.13	1											
	2											
	3											
	4											
	5											
2003.07.25	1											
	2											
	3											
	4											
	5	V										
2003.07.28	1											
	2											
	3											
	4											
	5											
2008.04.09	1			R		V					S	
	2					V				G		
	3		T	R	I	V			E		S	
	4					V					S	
	5				I	I		P			S	
2008.04.23	1			R		V				G	S	
	2		T	R			E			E		
	3				I	I					S	
	4		T	R			E			E	S	
	5			R		V					S	P

**Table 3:** Characterization of HBV RT sequences

		**45**	**91**	**117**	**122**	**133**	**137**	**139**	**144**	**154**	**159**	**184**	**185**	**214**
		**T**	**H**	**Y**	**N**	**V**	**L**	**Y**	**W**	**V**	**K**	**R**	**F**	**A**
2003.07.13	1													
	2													
	3													
	4						S							
	5			H										
2003.07.25	1											G		
	2		R											
	3													
	4													
	5													P
2003.07.28	1							H						
	2													
	3													
	4													
	5													
2008.04.09	1	S				G								
	2	S				N				A				
	3	S			D	G								
	4					G								
	5				D	S					R			
2008.04.23	1								G					
	2				D	G								
	3				D	G								
	4								G					
	5	S				N							S	

## Discussion

In the present case anti-HBc was the only detectable HBV marker (“anti-HBc only status” [15,16]) between October 2003 and October 2007. The HBV DNA fluctuated between 10 to 357 IU/ml, indicating an occult chronic HBV infection. Several explanations have been declared for ‘anti-HBc only’ status, including false positive detection of anti-HBc, low replication level of HBV, HBV mutations, coinfection with other viruses and formation of HBsAg-anti-HBs immune complexes [17]. Anti-HBc only serostatus has been described frequently among individuals coinfected with HIV or hepatitis C virus (HCV) infection [18,19]. It is advised to monitor such patients at regular intervals by HBV serology [15,20] and the HBV DNA should be investigated in case of elevated transaminases [18].

Although about 17-42% of patients coinfected with HIV/HBV have anti-HBc only, there are few reports on the reactivation of HBV in these patients [20,21]. Previously, Chamorro et al. reported on a reactivation of HBV after the removal of lamivudine in an HIV-infected patient with anti-HBc as the only serological marker [22]. In the present case, the reactivation of HBV occurred spontaneously in a patient with a stable CD4+ T cell count who had not received antiretroviral drugs for years before the hepatic flare. An interesting finding is that the K to R mutation at s122 occurred during the flare-up phase and led to the change of the HBV serotype from adw to ayw. Most of the other aa substitution in the HBsAg found during the flare-up phase described here have been reported before and in combination with the K122R mutation, possibly causing HBV escape from anti-HBs. Amino acid substitutions, which change the hydrophilicity, the electrical charge or the acidity could change the conformation of the a-determinant [23]. M133I and D144E mutation were reported to cause a reduced HBsAg affinity to anti-HBs [24,25]. F134V mutation was supposed to be a vaccine induced escape mutant [26] and L175S mutation was found in patients with HBsAg-anti-HBs coexistence [27]. The replication competence of HBV strains from different time points need to be compared.

The question was raised whether the hepatitis flare in this patient could be interpreted as a superinfection. However, this patient was constantly positive for HBV DNA for many years. The sequence analysis revealed that the HBV preC/C region was strictly conserved during the course of infection, an evidence preferably for reactivation. Another point for dispute is the fact that there was no obvious pressure by anti-HBs responses for the selection of escape mutations. However, Weinberger et al. [28] indicated that the variability of the major hydrophilic loop of HBsAg was raised significantly in individuals with anti-HBc only compared with HBsAg positive individuals. The patient in this study was positive for anti-HBc only for a long period and represents a typical case of such status. The anti-HBc only status during the chronic phase might be due to the control of HBV replication by the immune surveillance and/or due to the detection escape mutation that may occurred early in the chronic phase. The mutations may have accumulated under these pressures and became the dominant strain. In addition, it is possible that the patient had a low anti-HBs response which remained under the detection limit due to HIV infection. We also detected anti-HBc IgM in the patient during chronic HBV infection and in the flare-up phase. Although normally the appearance of anti-HBc IgM indicates a new infection, it may also be detected during HBV reactivation [29,30]. Therefore, we could not completely rule out the possibility of superinfection but do not have evidence favoring this hypothesis.

Before the hepatitis flare, the patient did not receive any antiviral treatment, immunosuppressive therapy, or radiation treatment, which may be related to HBV reactivation [31,32]. It seems that the reactivation happened spontaneously. The accumulation and selection of HBV escape mutants might be one important factor. On the other hand, the immune suppression caused by HIV infection probably also played a role in HBV reactivation. Previously, interleukin-6 (IL-6) was proven to serve as the main bystander mediator of radiotherapy induced HBV replication and IL-6 and radiotherapy have synergistic effect [33]. However, it is not clear whether the cytokine profile was changed in this patient and related to the reactivation.

In conclusion, we reported on a case of spontaneous HBV reactivation in an HIV coinfected patient with isolated anti-HBc, in which the escape mutants in s gene might be responsible for the flare-up. Continued monitoring of the patient with respect to HIV and HBV is necessary for recognizing a possible re-flare of the HBV infection. Retrospectively, the reactivation of HBV might already have been diagnosed in August 2007 when HBsAg and HBV DNA tested positive as indicated from Table 1.

## Consent

A copy of the written consent is available for review by the Editor-in-Chief of this journal.

## Competing interests

The authors declare that they have no competing interests.

## Authors’ contributions

RJP carried out the molecular genetic studies, participated in the sequence alignment and drafted the manuscript. SG, JV and SE provided the clinical data and revised the manuscript. XWC and MJL conceived of the study, and participated in its design and coordination and helped to draft the manuscript. All authors read and approved the final manuscript.
